# Routine Manipulations and Operations from the Stand-Point of Asepsis and Antisepsis1Read at the thirty-sixth annual meeting of the American Veterinary Medical Association, September, 1899.

**Published:** 1900-02

**Authors:** Wm. Herbert Lowe

**Affiliations:** Paterson, N. J.; Formerly United States Veterinary Officer of the Port of New York


### ROUTINE MANIPULATIONS AND OPERATIONS FROM THE STAND-POINT OF ASEPSIS AND ANTISEPSIS.[1]

BY WM. HERBERT LOWE, D.V.S.,
PATERSON, N. J.
FORMERLY UNITED STATES VETERINARY OFFICER OF THE PORT OF NEW YORK.

(Continued from page 7, January, 1900.)

IN addition to the resources of cleanliness for preserving wounds from becoming the seat of the vital activity of microörganisms, there still remains to the veterinary surgeon the employment of direct applications to wound-surfaces of substances which have the power either to destroy them outright or to restrain their growth. These agents are classified as antiseptics, and their use as above constitutes wound disinfection.

The possibility of obtaining antiseptic results in a wound by agents that simply restrain the growth of septic organisms as well as by those that destroy them, is a matter of great practical importance, for it has increased the number of substances available for antisepsis, and since the preventive effects of many agents can be accomplished by much smaller amounts than their destructive effects, it has been found possible to obtain their antiseptic effects with less local irritation of the wound itself and less liability of danger from absorption of poisonous quantities of the agent into the blood.

In estimating the usefulness of any agent as an antiseptic application in the treatment of wounds, three things have to be taken into consideration :

1. Its power as a bactericide or as a restrainer of bacterial multiplication.
2. Its immediate local effect on the wound-surfaces--neutral, irritant, or caustic.
3. Its remote constitutional effect when absorbed into the circulation.

The bacteria of different species manifest different degrees of vital resistance to chemical reagents.

[1] Read at the thirty-sixth annual meeting of the American Veterinary Medical Association, September, 1899.

The following table exhibits the strengths needed to destroy the vitality of the micrococci of pus, or to prevent the development of the organisms. The practical application has found its place rather in the preliminary disinfection of materials likely to come in contact with wounds in the course of formal surgical operations than in applications to wounds themselves :

TABLE OF BACTERICIDAL STRENGTHS.

| Reagents | Efficient in the proportion of one part in |
|---|---|
| Mercuric bichloride | 20,000 |
| Potassium permanganate | 833 |
| Iodine | 500 |
| Creosote | 200 |
| Sulphuric acid | 200 |
| Carbolic acid | 100 |
| Hydrochloric acid | 100 |
| Zinc chloride | 50 |
| Tinctura ferri chloridi | 25 |
| Salicylic acid dissolved by sodium borate | 25 |
| Citric acid | 8 |
| Chloral hydrate | 5 |

The following table shows the minimum quantity required to prevent the development of the pyogenic micrococci :

TABLE OF STRENGTHS REQUIRED TO RESTRAIN BACTERIAL MULTIPLICATION.

| Reagents. | Efficient in the proportion of one part in |
|---|---|
| Mercuric bichloride | 35,000 |
| Iodine | 4,000 |
| Sulphuric acid | 1,800 |
| Carbolic acid | 500 |
| Salicylic acid and sodium biborate, equal parts | 200 |
| Boric acid | 200 |
| Ferric sulphate | 200 |
| Sodium biborate | 100 |
| Alcohol | 10 |

The foregoing tables are from Dr. L. S. Pilcher's work on *The Treatment of Wounds*, recently issued. I am also indebted to the same author for much valuable data in connection with the subject under consideration.

Comparison of the two tables shows that the more potent bactericides have the power of resisting multiplication in quantities considerably less than are required to destroy vitality. In the case of iodine, the difference is eightfold; in that of carbolic acid, fivefold; in that of sulphuric acid, fourfold, etc.

In addition to those soluble agents marked antiseptic power attaches to many substances which are insoluble or comparatively so. These include iodoform, naphthaline, zinc oxide, bismuth subcarbonate, subnitrate, suboxide and subgallate, aristol, ariol, iodol, nosophen, and other similar compounds. The common property of this group of agents is the inhibition by their presence of bacterial growth and multiplication. To some of them attaches also a chemotatic power—that is to say, the power to stimulate the accumulation of living energetic cells in the wound-surfaces and borders. The effect of this is to strengthen the local resisting power of the tissues and to favor rapid repair.

Carbolic acid, the agent first used in the application of modern antisepsis, and used so extensively by Lister, is now largely replaced by other agents. The properties that commend carbolic acid for use as an antiseptic are its reliability and its diffusibility. Its disadvantages are the local irritation which it excites, its volatility lessening its usefulness as an agent to secure permanent antisepsis, making frequent renewals of the dressings necessary, which renewals violate another fundamental principle of wound-treatment, that of rest; its toxic qualities, dogs and cats being particularly susceptible. Carbolized ointments and solutions of carbolic acid in oil or alcohol have been shown by Koch to be absolutely inert in respect to their action on bacterial life, either on the spores or the fully developed organisms. Anthrax spores introduced into oily solutions of carbolic acid, of thymol, and of salicylic acid, in each at the end of three months were still found capable of development. When, however, the oily solution comes in contact with substances containing water it undoubtedly gives up part of the acid to these, and in this way antiseptic effect may be produced. Carbolic acid is used to good advantage in the antiseptic bath for the immersion of metallic instruments.

Creolin, cresol, and other products of coal-tar distillation have largely superseded carbolic acid. These substances have marked antiseptic power, do not produce so much local irritation, and are less toxic. They form milky emulsions when mixed with water, and have been used in such emulsions in from 2 to 5 per cent. strength.

Salicylic acid and boric acid are feeble bactericides, but potent as restrainers of bacterial activity. They are devoid of the irritating and poisonous effects of corrosive sublimate and carbolic acid.

Corrosive sublimate is largely used as an antiseptic. Koch found that the spores of the bacilli of anthrax, though unaffected by other antiseptics, were killed in a few minutes by a solution of corrosive sublimate, 1: 1000, and to have been prevented from developing by a solution of 1: 5000. The necessity for changes of dressing with corrosive sublimate are rare. The danger of poisoning from absorption is slight when in the hands of a competent practitioner. Corrosive sublimate is the most reliable agent available for the disinfection of fresh wounds that are known or presumed to be infected. For this purpose 1: 5000 aqueous solutions are used. It is much used also in the strength of 1: 1000 or 1: 2000 for the disinfection of the skin in regions that are to be operated upon, and for the disinfection of the hands of surgeons and assistants.

*Iodine and Iodoform.* The antiseptic properties of iodine are manifoldly greater than those of carbolic acid—as a bactericide five times, and as a restrainer of bacterial growth eight times as great—but it has attracted general attention only since the introduction into use as an antiseptic of its compound, iodoform. Iodoform did not irritate the wound ; it was used both as an application to the wound-surfaces and as an external, antiseptic, protective dressing ; its decomposition or its volatilization was so slow that the frequency of the dressings required was greatly lessened. The odor and the toxic qualities of the drug were the chief disadvantages that limited its use. It diminishes secretion, decomposes ptomaïns, and stimulates leucocytosis, and when itself decomposed liberates the germicidal iodine. Aristol, airol, iodol, and nosophen are among the other iodine compounds used as substitutes for iodoform.

*Salts of Bismuth.* Bismuth subnitrate and bismuth subgallate are of value, especially when used as a powder to be dusted over the skin surrounding a wound, to restrain the activity of epidermal organisms and as a powder applied to granulating surfaces to promote its cicatrization.

*Salts of Zinc.* Zinc oxide is especially worthy of mention and commendation as a dusting powder in the treatment of burns, excoriations, and abrasions, and for use upon the skin and about a wound and along a suture line. It restrains the activity of the epidermal organisms. It is in itself perfectly unirritating, and with serous secretions forms a bland and soft magma. A mixture of equal parts of zinc oxide and of water, with the addition of 10 per cent. of zinc chloride, forms a paste which, when applied to wounds, rapidly dries into a firm protective crust or artificial scab.

Zinc chloride is very soluble in water, and in the stronger solutions is perfectly caustic. A strength of 1 : 50 is required to destroy pyogenic organisms, and in this strength the living tissues are affected by it, a zinc albuminate being formed which appears as a white translucent film covering the surface to which it has been applied. Its use is restricted to the disinfection of wounds that are already frankly septic. Solutions of from 5 to 8 per cent. strength should be used. The film of zinc albuminate which results is markedly resistant to new bacterial invasion, and further restrains exudation and protects mechanically the underlying tissue. This combination of qualities makes it an antiseptic agent of the greatest value in veterinary practice in dealing with sores and open infected wounds.

Among antiseptic agents may be mentioned aluminum acetate, acetanilid, hydrogen peroxide, mercuric iodate, naphthalin, beta-naphthol, pyoktanin, formic aldehyde, and heat.

*Heat.* The temperature of boiling water, 212° F., is sufficient to quickly destroy all known pathogenic bacteria. Most spores are also destroyed by this degree of moist heat, but so marked a power of resistance inheres in some spores that they are not destroyed by even so long an exposure as two hours. For ensuring the absolute destruction of all spore life the method of *fractional sterilization* is available.

None of the ordinary pus-forming bacteria are spore-bearers, so that their destruction in such dressings and instruments and materials to be used in wound treatment as will not be injured by moderate heat is simple and rapid. When microörganisms in a desiccated state are exposed to dry heat a much higher temperature is required for their destruction than when they are moist or when they are exposed to the action of hot water or moist steam. For destroying the ordinary pyogenic organisms exposure to at least 212° of dry heat for one hour is required, but to ensure the destruction of spores an exposure of three hours or more to a temperature of 284° F. is necessary.

The possibility of using heat conveniently in the shape of boiling water or of steam for the absolute and certain destruction of bacteria has caused these agents to very largely supersede chemicals for the disinfection of instruments, dressings, and many of the accessories used in the treatment of wounds. Thermic sterilization is much more readily and certainly accomplished than is chemic sterilization.

*The Dressings.* For absorbent and protective purposes the chief part of a wound dressing is composed of suitable pieces of loosely woven, thin, gauzy cotton cloth, similar to the "cheese-cloth" or "butter-cloth" of the shops; this constitutes the "gauze" of the surgeon. Other absorbent materials may be substituted according to the convenience of the particular case, such as pads of moss or of sawdust, masses of the cotton fibre known as engineer's waste, or of soft absorbent paper, or quantities of oakum, jute, or "wood-wool;" but the superior convenience and the unequalled absorbing qualities of the gauze have caused it to be accepted almost universally by practitioners of human surgery as the staple dressing material. It also has an important place in surgical dressing in veterinary practice. This dressing, like all other dressings and everything else, requires sterilization. Steam sterilization is convenient and effectual.

*Boro-salicylated Moist Dressings.* For the absorption of primary wound discharges a moist dressing is superior, and in view of the imperfect character of all skin sterilization it is always more prudent to have the dressing which comes in direct contact with the skin in the vicinity of a wound moistened with a solution which at least shall inhibit bacterial activity. Corrosive sublimate solutions at once suggest themselves on account of their potency as a bactericide, but they are too irritating to the skin to be left on many hours in contact with it, and if used freely are liable to provoke the poisonous effects of the absorption of the drug. Carbolic acid is equally objectionable. Boric and salicylic acids, feeble bactericides but potent as retainers of bacterial activity, are free from the objections named as to corrosive sublimate or carbolic acid, and may be used in a solution made by dissolving one ounce of boric acid and eighty grains of salicylic acid in a gallon of boiled water for the preparation of a moist antiseptic absorbent dressing.

Cotton-wool, tow, oakum, jute, turf moss, sawdust, and many other materials are used for surgical dressings. They can be impregnated with such antiseptics as the surgeon considers indicated. In veterinary practice the animal must be tied or secured so that he cannot bite or tear the dressings and bandages off.

I trust that I have said enough to inspire veterinarians to be more painstaking in their routine manipulations and operations. If there is any part of the routine practice that veterinarians are remiss in it is in carrying out the principles of aseptic and antiseptic surgery. The same principles apply to major surgery, and the practitioner who applies these in his everyday practice will be able to apply them also and obtain better results in such major operations as he has to perform.

We want in the veterinary profession to-day men with common sense, common honesty and ability, and a disposition to do the ordinary routine practice methodically and well, who will practice upon the latest scientific aseptic and antiseptic methods ; men who are willing to confine their efforts to the legitimate limits of the profession ; men who are horsemen in the highest sense of the term, but who are not horse-dealers, horse-jockeys, or liverymen ; men who are working to earn all the money they can legitimately, but who would not sell their birthright for a "mess of pottage."

